# Victims, offenders and victim-offender overlaps of knife crime: A social network analysis approach using police records

**DOI:** 10.1371/journal.pone.0242621

**Published:** 2020-12-11

**Authors:** Laura Bailey, Vincent Harinam, Barak Ariel

**Affiliations:** 1 Institute of Criminology, University of Cambridge, Cambridge, United Kingdom; 2 Institute of Criminology, Faculty of Law, Hebrew University, Jerusalem, Israel; London School of Economics, UNITED KINGDOM

## Abstract

Knife crime is a source of concern for the police in England and Wales, however little published research exists on this crime type. Who are the offenders who use knives to commit crime, when and why? Who are their victims, and is there a victim-offender overlap? What is the social network formation for people who are exposed to knife crime? Using a multidimensional approach, our aim is to answer these questions about one of England and Wales’ largest jurisdictions: Thames Valley. We first provide a state-of-the-art narrative review of the knife crime literature, followed by an analysis of population-level data on central tendency and dispersion of knife crimes reported to the police (2015–2019), on offences, offenders, victims, victim-offender overlaps and gang-related assaults. Social network analysis was used to explore the formations of offender-victim networks. Our findings show that knife crime represents a small proportion of crime (1.86%) and is associated largely with violence offenses. 16–34 year-old white males are at greatest risk of being the victims, offenders or victim-offenders of knife crime, with similar relative risks between these three categories. Both knife offenders and victims are likely to have a criminal record. Knife crimes are usually not gang-related (less than 20%), and experienced mostly between strangers, with the altercation often a non-retaliatory ‘one-off event’. Even gang-related knife crimes do not follow ‘tit-for-tat’ relationships—except when the individuals involved have extensive offending histories and then are likely to retaliate instantaneously. We conclude that while rare, an incident of knife crime remains predicable, as a substantial ratio of offenders and victims of future knife crime can be found in police records. Prevention strategies should not be focused on gang-related criminals, but on either prolific violent offenders or repeat victims who are known to the police—and therefore more susceptible to knife crime exposure.

## 1. Introduction

Reports of deaths arising from knife crimes have dominated United Kingdom (UK) headlines for the past half-decade, with U.S. President Donald Trump comparing the UK to a ‘military war zone’ [1]. British politicians have declared a ‘national emergency’, promising to ‘come down hard’ on the ‘scourge’ of knife crimes [2, 3]. Yet, the literature on the topic is, interestingly, scant [4].

As Eades et al. [5] contended, in order to curb knife crime we must first understand what it is, who is involved and why certain individuals are attracted to it. Furthermore, while knife armament is seen as a relatively recent crime trend (it is not), the causes cited by those involved bear resemblance to the criminological literature on the victim-offender overlap (V-OO), where victims and offenders, based on shared characteristics and social space, are ‘one-in-the-same’ [6, 7]. This suggests that V-OO may be a viable theoretical composition through which we can contextualise, forecast, and potentially prevent knife crime.

While V-OO provides an important assessment of individuals and their risk factors, our understanding of knife crimes can be aided by a more holistic view: explicating all the actors involved in knife crime, and only then reviewing their social ties. In this sense, social network analysis (SNA) provides an opportunity to look at the interdependencies between individuals involved in knife crime [8]. As noted by Lauritsen and Laub [9], it is not only an individual’s victimisation that needs to be considered, but also the victimisation within their social network, which can potentially be used to determine risk of both future victimisation and offending. For this, however, we must assume there is an organisational or structural component—i.e. gangs or organised crime—that causes knife-enabled violence to proliferate and spread through networks: violence begets violence, and proximity to violence places an individual at greater risk of victimisation [10]. This, however, has not been addressed in the violence literature.

By combining V-OO and SNA, this study provides insight into the nature of knife crime and how it spreads, if at all, among individuals and their criminal associations. In particular, SNA allows for the examination of the network structure of knife crimes in Thames Valley. This offers key strategic insight which may inform future law enforcement interventions. Importantly, this study also examines the role of retaliation and inter-gang violence in knife crime. We do this by examining population-level data of all non-domestic knife crimes recorded by the Thames Valley Police (TVP) between 2015 and early 2019.

## 2. Literature review

### 2.1 Difficulties in quantifying knife crime in the UK

Based on police records, knife crime occurred in less than 3.3% of all violent and weapon crime in 2019, which makes it a rare event [11]. Regardless of its statistical rarity, knife crimes are serious events where those involved may be seriously harmed. As such, further research into its occurrence and composition may yield insight into reducing harm. Longitudinally, the Office of National Statistics report that knife crime in the UK has risen by 7% between 2018 and 2019 [12]. However, the rate of growth is slowing when data are analysed for a more extensive period of time [13]. As it is often the case with low incidence events, longitudinal analyses can produce dramatic year-on-year fluctuations, though, in relative terms, the variations represent only small changes in incidence counts [14, 15]. This may drive the dramatization in the media over the recent reported ‘spike’ in knife crime [16–19].

Another issue is whether police records are representative of ‘true population means’. First, modifications in crime recording practices could be driving these supposed increases in knife crime [12, 20]. There are variations between police forces, between police units and over time [21], which makes both the validity as well as the reliability of ‘official statistics’ suspect at times [22, 23]. Second, as observed by Ariel and Bland [24], police proactivity can be the main driver for reported increases in knife crime, especially when ‘knife crime’ includes possession offences, which are directly affected by targeted police activity such as stop and search [25]. Indeed, the combination of surge funding, associated stop and searches increases, and the explicit focus on weapons will affect the validity of police-recorded data. Moreover, this distinction between possession offences and violent crimes precipitated with a knife should be separated when we analyse data trends—both longitudinally and cross-sectionally.

#### 2.1.1 Who is more likely to commit a weapon offence?

Just like the definition of what constitutes a knife crime, the antecedents for carrying or using a sharp blade are diverse. However, the literature on the perpetrators of knife crime is scant, with much of the knowledge arising from general studies of violence in which the data on weapon offences are often auxiliary to the main research questions. Still, the available evidence remains informative, and unravels several themes that cut across jurisdictions, contexts and time.

Research shows that young males are substantially more likely to be involved in knife crime [26 p7; 7]. The typical age range of those involved is between 13 and 24 years [27], which mimics the age-crime curve in terms of violence more broadly. If the study’s estimates are valid, then this translates to nearly 18,000 boys in this age group who carry a weapon [21]. The Serious Violence Strategy [21] further revealed that primarily underprivileged children, or those with four or more siblings, are more likely to be involved in serious violence, which illustrates the importance of studying familial links in violence.

Knife crime is more likely to take place in and around schools [28]. A recent ‘Freedom of Information request’ by the BBC News has indicated that out of 27 police forces in England and Wales that provided data, weapon possession (primarily sharp objects) at schools had doubled in four years. However, in absolute figures the rise consists of an increase from fewer than 500 per year to over 950 weapon possession offences in schools. Finally, one report suggests that carrying a weapon in school is associated with underachievement amongst 10 to18-year olds and more profoundly with school exclusions (but not due to weapon possessions) and a higher proportion had been persistently absent from school [29].

### 2.2 The victim-offender overlap and its pertinence to knife criminality

The victim-offender overlap (V-OO) is well established within criminological research [30, 31]. The V-OO was first presented by Von Hentig [32] and later put forward by Wolfgang [33] and Gottfredson [8]. The authors identified a crossover between victims and offenders, determining three categories of criminally involved individuals: ‘pure victims’, ‘pure offenders’, and those who are ‘one-and-the-same’ (or victim-offenders). These ‘victim-offenders’ often share social characteristics, risk factors and etiological linkages which are different from pure victims and offenders [34].

Several theories have been proposed to explain the V-OO. These can be grouped into two schools of thought: the dynamic causal perspective and the population heterogeneity perspective [see review in 35]. First, the dynamic causal perspective purports that when the likelihood of being victimised increases, there is an immediate elevated risk of committing a violent offence [36]. In a longitudinal study of Swiss youth, Averdijk et al. [37] found that following victimisation, individuals are less likely to feel shame or remorse when committing a future offence—which tends to justify subsequent crime. On the other hand, the population heterogeneity perspective argues that victimisation and offending do not cause one another but are instead caused by stable personality characteristics or environmental factors that precipitate both [35]. The general theory of crime is an example of this view, stipulating that low self-control is the cause of criminality [38]. Whether this arises from an offender’s prior criminal history [39] or the history of their peers, the risk of victimisation is more pronounced [40, 41]. For example, in a study of victims with low self-control who were subject to a serious assault, Singer [42] identified that these individuals had the highest probability of having a friend arrested, belonging to a gang, using a weapon and committing a serious assault. Thus, a third factor causes both the likelihood of offending as well as being a victim—but there is no necessarily reciprocal relationship between the two; the end result, however, is the same: a V-OO.

Gangs have received specific attention in the V-OO research. In 2009, Melde et al. [43] conducted a study of youths aged between 10 and 16 years, identifying that those in gangs report greater levels of actual and perceived risk of victimisation compared to non-gang-members. In a longitudinal study of Chicago youth, Zimmerman et al. [44] looked at the relationship between victims and offenders, finding that the odds of offending are 76% greater if victimised, and 70% greater if victimised by a gang member. Papachristos et al. [10] contend that gang membership is associated with a tripled risk of violent victimisation. Similarly, Zavala [45] found that gang membership increases the risk of victimisation by 447%, and offending risk by 249%. Given this body of evidence, the underlying assumption ought to be that V-OO is ubiquitous, and that knife crime is likely to follow suit, although to the best of our knowledge no published research has looked at this phenomenon specifically in the context of knife crime.

### 2.3 Social network analysis and violent crime

Criminal conduct is a social behaviour, with most offenders being embedded within networks of delinquent and criminal friends [46]. The examination of these social networks can also provide insight into crime patterns and structures [47, 48]. In recognising the connectivity between individuals, SNA allows for the examination of the connective properties that link these individuals and influence their behaviour [49].

Haynie [50] illustrated that the more delinquent peers central to one’s network, the more delinquency will be measured at the level of the individual. The density of a network is also key, whereby a greater number of actors are said to increase the victimisation and offending risk [38]. In a study looking at juvenile co-offending networks, McGloin and Piquero [51] found that members of a ‘dense’ network are often specialised in their criminality when offending with others, while sparse networks demonstrated less criminal specialisation. Therefore, those who regularly offend together, such as gang members, will often commit similar offences due to the lack of skills and diversity afforded by group members.

Subsequently, the proliferation of violence is a key focus within SNA, often concluding that violence is infectious and passes through social networks [52–54]. By using dyadic analysis, Papachristos [55] identified that murders between rival gangs are best understood as social contagion, carried out within organised networks, rather than committed at random [56]. This is supported by the finding that 70% of victimisations occur within recognisable social networks; even indirect exposure to victimisation increases the likelihood of victimisation [10].

In relation to knife crime, similar findings were described as a ‘replicative externality’, whereby knife criminality proliferates though a person’s social network [57]. This highlights the importance of understanding an individual’s network and, in particular, their associations and connections, given the increased likelihood of offending being committed within that network.

Admittedly, most SNA studies on violent networks have been conducted in the US, with an underrepresented crime portfolio from UK gangs [58]. Therefore, whilst being able to draw conclusions on the potential validity of SNA to examine the proliferation of knife crime within the UK, these results might be limited in their generalisability as to whether knife crime mimics the contagion effect of U.S. gun and gang violence.

## 3. The present study

Given the literature review, this research aims to address two research questions:

1. Who offends and who is victimised by knife crime, and is there evidence of V-OO?
2. What is the network structure of knife crimes in Thames Valley, based on these police records?

## 4. Data and methods

### 4.1 Data

To address the research questions, our study uses a population-level dataset, covering the full cohort of knife crimes and linked individuals across the geographical area serviced by the Thames Valley Police (TVP) of England and Wales. TVP is the largest non-metropolitan police force in England and Wales, with a jurisdiction over 2,200 m^2^, three primary counties (Buckinghamshire, Berkshire and Oxfordshire), a diverse population of more than 2.34M residents and approximately 6M visitors who come to the Thames Valley each year. The Thames Valley is located in the southeast region of England, west of London. The police force area is divided into 12 Local Policing Areas, with more than 4,120 frontline officers, 3,400 police staff and more than 500 volunteers.

The dataset used originates from police-recorded information reported and retained in local police records referred to as the Records Management System that is used by the TVP (RMS Niche), which manages information in relation to people, locations, organisations and incidents. The dataset contains all non-domestic knife crime occurrences across the TVP Force Area (covering the entirety of Oxfordshire, Buckinghamshire and Berkshire) reported to or by the police within a four-year period (01/04/2015 to 31/03/2019). This four-year dataset was selected due to changes in knife crime recording practices prior to this period. Importantly, we define ‘knife crime’ as any event where a knife, sharp instrument or other bladed implement was present or used to assault another person.

For the period of interest, a total of 515,939 unique non-domestic crimes were reported (as an illustration, in 2019 199,772 crimes were recorded, of which 58,752 violent crimes and 1,627 robberies were registered. However, only 10,146 non-domestic knife crime offences were identified based on RMS Niche filters, which recorded the presence or use of a knife during the commission of an offence. Of these 10,146 knife crime offences, 10,099 unique cases can be identified (i.e. 47 duplicate entries were removed). This constitutes 2.2% of all offenses in Thames Valley over the period of study. These offences occurred against 6,896 unique victims and 7,231 unique offenders who were identified. For the purposes of this study, “offender” is defined as an individual who initiates a knife crime while “victim” is defined as an individual who is on the receiving of the knife crime within that specific knife crime incident. These definitions are reflective of how victims and offenders were categorized within the dataset by Thames Valley crime analysts and database managers. With respect to the V-OO, it is important to clarify that an individual can be a victim in one knife crime event and an offender in another separate event.

In terms of data points, we gained access to anonymised information on all the individuals involved, including whether they are affiliated with gangs or organized crime groups (OCGs); their gender, ethnicity, date of birth, prior offences and victimisations; and all the quantifiable information on system. Importantly, 610 unique individuals were identified as both a knife crime victim and offender (V-OO), representing 8.8% of the victim population and 8.4% of the offender population.

### 4.2 Analysis plan

#### 4.2.1 Descriptive statistics

Measures of central tendency and dispersion were used to summarize the 10,099 knife crimes. These basic descriptors are useful for understanding both the socio-demographic and spatial composition of knife crime, as well as the presence of a V-OO.

#### 4.2.2 Social network analysis

We employed descriptive network analysis to illustrate how the social networks of knife crime offenders are constructed in Thames Valley. All network statistics and modelling were conducted in R using the *igraph* [59] and *statnet* [60] packages, while visualizations were conducted in Gephi. Social network analysis offers unique insight into the composition of a criminal network. Moreover, by examining the global structure of knife crimes, we can better diagnose the UK’s perceived knife crime epidemic and offer policy recommendations on how to deal with it. As such, social network analysis represents a novel means by which UK knife crimes can be examined.

From the 10,099 identified knife crime offences, two networks were created: 1) a one-mode ‘actor-to-actor’ network featuring offenders and victims and 2) a one-mode ‘actor-to-actor’ network featuring offending OCGs and victimised OCGs. Given the strict requirement of both an identifiable offender *and* victim, only knife crime offences with both a known offender and victim were used to construct these networks. Offenders and victims were identified based on their Unique Nominal ID and criminal association. As some offenders and victims could not be linked to a victim or offender, respectively, due to data limitations they were removed from analysis. As such, the offender-victim network consisted of 7,044 pairs, with 5,020 unique offenders and 4,550 unique victims. The OCG-to-OCG network consisted of 208 pairs, with 97 unique offending OCGs and 86 unique victimised OCGs. A link between the nodes was defined as co-offending or involving knife crime [61].

More formally, our analyses use directed ties, meaning that the tie from *i* to *j* is not necessarily reciprocated (*i* may attack *j* with a knife, while *j* may not do the same to *i*). An interaction between an offender and victim is regarded as a tie, *y*_*ij*_, where *y*_*ij*_ = 1 if a violent interaction has occurred between offender *i* and victim *j* and *y*_*ij*_ = 0 if not. As our data are weighted, *y*_*ij*_ may be greater than 1 if offender *i* and victim *j* have had multiple interactions.

We applied four network measures to assess the structure of these networks: network density, average in/out-degree centrality, reciprocity and modularity. Density measures the overall interconnectedness of a network (e.g. the number of possible interactions between offender *i* and victim *j*). To this extent, network density measures the total number *i* to *j* ties divided by the number of possible ties in the network. Represented by a coefficient ranging from 0 to 1, a high density (close to 1) indicates that offenders tend to engage in knife crimes frequently and with different victims. Conversely, a low density (close to 0) indicates that offenders tend to assault a single victim on an infrequent basis.

Average in/out-degree centrality measures the average number of unique ties an actor has within their respective network. For our purposes, it indicates the average number of unique victims an offender has (outdegree centrality) and the average number of unique offenders a victim has (indegree centrality). Reciprocity delineates the proportion of mutual connections in a directed graph [62]. It is commonly defined as the probability that the opposite counterpart of a directed edge is also included in the graph. That is, the proportion of *i* to *j* ties divided by the total number of ties in the network. Reciprocity indicates the extent to which offenders are victimised by their previous victims.

Finally, modularity indicates the extent to which a network decomposes into modular communities [63]. In the network sciences, a community is comprised of nodes that are more densely connected to each other than to other nodes in the network. For our purposes, modularity reflects the number of knife crime communities within the network. To this extent, the number and connectivity of isolated knife crime communities serves as an indicator of the level of ‘information flow’, and by extension, fragmentation, within the network. Modularity is a chance-corrected statistic ranging from -0.5 to 1. It is defined as the fraction of ties that fall within the given groups minus the expected fraction if ties were distributed at random. A high modularity score (Q) suggests that the network is compartmentalized into distinct subgroups [for further technical details see 64].

### 4.3 Data limitations

Perhaps the foremost limitation of any police data system is the poor validity of the information recorded therein [65]. Fluctuations in criminality due to police proactivity and the quality of reporting are an open concern [24]. Therefore, this study only presents data on what TVP know, which likely masks the true population figures: official statistics are unlikely to represent the true scale of knife criminality across Thames Valley.

In terms of data quality, we lack any information on *how* knives are used. Without knowing this, it is difficult to measure the true harm arising from knife crimes, without manually reviewing all 10,099 knife crime offences—an exercise that was beyond the scope of this inquiry.

Finally, while knife crimes may be underreported, known knife crime offenders may be over-reported. As law enforcement often suspect those already known to them of committing a crime [54], the number of times that an offender is linked to multiple knife crimes may be inflated.

## 5. Results

### 5.1 Descriptive statistics for knife crime trends, offenders, and victims

#### 5.1.1 Knife crimes by year and crime sub-classification

**Fig 1:**
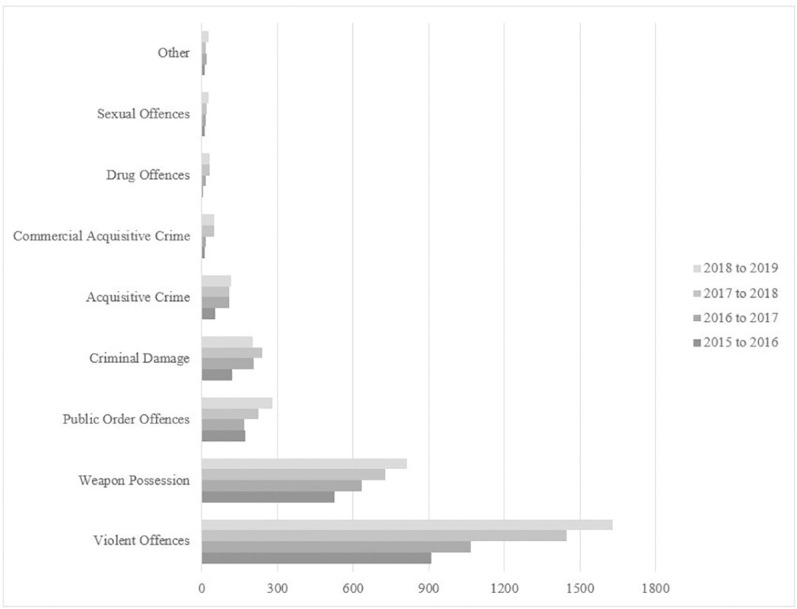
Count of all knife crime offences based upon crime sub-classification by year.

During the four years under review, the proportion of all non-domestic crime represented by knife crime increased by an average of 1.86%, while the rate of all non-domestic crime increased by an average of 6.52% each year. However, despite the increasing volume of knife crime each year (2015/2016: 1,816; 2016/2017: 2,244; 2017/2018: 2,867; 2018/2019: 3,172), the rate of growth has been inconsistent, with a 27.8% increase from 2016–2017 to 2017–2018 and 10.6% increase in 2018–2019 from the previous year. Importantly, when the data are disaggregated based on offence sub-classifications, we find that violence and weapons possession offences drive year-to-year knife crime increases (see Fig 1).

Of the 10,099 knife crime offences, 1,636 offences are linked to police-recognised organized crime groups (OCGs), which represents 16.2% of all knife crimes within the four-year period. To this extent, 236 offences involved both an OCG-affiliated offender and victim, while 1,380 and 492 offences involved only an OCG-affiliated offender and victim, respectively. Notably, of these 1,636 OCG-related knife crimes, 294 were linked to drug activity distributed between county drug lines and by local drug dealers.

#### 5.1.2 Socio-demographic characteristics of knife crimes

Within the 10,099 knife crime offences, there are 6,896 unique victims and 7,231 unique offenders. The key demographics are presented in Table 1. In general, we can see that White (Northern European) males between the ages of 16 and 34 constitute the majority of knife crime victims within Thames Valley. This is also the case for offenders, who are 87.6% male, 59.6% White (Northern European), and 59.3% aged 16 to 34.

**Table 1:** Key demographical characteristics for victims and offenders.

	Victims			Offenders		
Characteristic	Total	%	Range (*M*)	Total	%	Range (M)
*Gender*						
Male	5,112	74.1%	-	6,336	87.6%	-
Female	1,642	23.8%	-	945	13.1%	-
Unknown	142	2.1%	-	0	-	-
*Ethnicity*						
Asian	748	10.8%	-	706	9.8%	-
Black	549	8%	-	1,301	18%	-
Chinese, SE Asian, etc.	33	0.5%	-	20	0.3%	-
Middle Eastern	29	0.4%	-	24	0.3%	-
White—North European	3,789	54.9%	-	4,313	59.6%	-
White—South European	99	1.4%	-	152	2.1%	-
Unknown	1,469	23.9%	-	715	9.9%	-
*Age Ranges*						
All Ages	-	-	2–91 (31)	-	-	7–96 (26.5)
Males	-	-	2–91 (31)	-	-	9–96 (26.5)
Females	-	-	2–90 (33)	-	-	9–95 (29)
< 10 years	66	1%	-	31	0.4%	-
10 to 15 years	612	8.9%	-	988	13.7%	-
16 to 24 years	1,965	28.5%	-	2,580	35.7%	-
25 to 34 years	1,576	22.9%	-	1,705	23.6%	-
35 to 44 years	1,202	17.4%	-	1,030	14.2%	-
45 to 54 years	723	10.5%	-	586	8.1%	-
55 to 64 years	338	4.9%	-	194	2.7%	-
≥65 years	173	2.5%	-	63	0.9%	-
Unknown	241	3.5%	-	54	0.7%	-

Of the 6,896 unique victims and 7,231 unique offenders, 548 (7.8%) and 1,380 (19%) had a link to a recognized OCG, respectively. Furthermore, 38% (130) of OCG-affiliated victims were members of a drug dealing gang, with 87 and 52 of these victims linked to ‘county lines’ (County Line is a term used to describe gangs and organised criminal networks involved in exporting illegal drugs into one or more importing areas within the UK, using dedicated mobile phone lines or other form of “deal line”) and local drug dealers, respectively. Similarly, 34% (469) of OCG-affiliated offenders were linked to a drug gang while 28% (386) were affiliated with a weapons/firearms gang. Notably, 291 victims experienced more than one knife victimisation, accounting for 633 total knife crime offences. Furthermore, the majority of these victims were male (81.6%), White (Northern European) (52.5%), and aged 16 to 34 (55.4%).

#### 5.1.3 Habitual knife crime offenders and repeat victims of knife crime

Those identified as repeat victims and offenders both experienced, on average, two knife crime incidents. Of the 7,231 unique offenders, 1,564 (21.6%) committed more than one knife crime offence, accounting for 41.4% of all knife crimes within the four years of study. However, this distribution reaches a maximum of five incidents for victims and 13 for offenders. Importantly, incidents experienced by repeat victims (4.2%) represented a small percentage of all knife crimes (6.27%), while 21.6% of repeat offenders committed 41.4% of knife criminality.

Similar to repeat knife crime victims, repeat offenders were primarily male (92.5%), between the ages of 16 and 24 (42.4%), and predominantly White (Northern European) (57.3%), with Blacks and Asians accounting for 25.2% and 11.5%, respectively. To place these statistics in perspective, 8% Oxfordshire’s population are Black, Asian or other ethnic minorities (http://www.southoxon.gov.uk).

#### 5.1.4 V-OO

The data present evidence of a V-OO: 610 individuals can be identified as both a victim and an offender. This cohort accounted for 7.3% (734) of all knife crimes, with 410 offences committed by 145 repeat victim-offenders. Only nine knife crime incidents involved a victim and offender who were both repeat victim-offenders. In terms of gender distribution, 89% were male, comparable to all knife crime offenders within the dataset. White (North European) overrepresented, constituting 65.5% of victim-offenders, followed by Black (18.8%) and Asian (14.24%) victim-offenders. Moreover, the average age for male victim-offenders is 27 years and 29 for females (63.1% (385) are between the ages of 16 and 34).

In terms of repeat involvement in knife crime, victim-offenders are split into two groups: victimised and offending. Eleven per cent of victim-offenders experienced 22.9% of victim knife crime (1.9% of total knife crime), while 23.7% of victim-offenders committed 55.8% of victim-offender associated knife crime (4% of total knife crime). Of the 610 victim-offenders, 30.5% (186) were affiliated with an OCG. Of the 10,099 unique knife crimes, only 236 offences involved victim-offenders with a known affiliation to OCGs and gangs (2.34%). Groups of offenders that deal drugs represent more than 33% of all OCGs and gangs. Within this group of victims-offenders, 47% are linked to country lines, 32.9% linked to local drug dealers, and the rest (20%) to both. Of the 610 unique victim-offenders, they experienced 679 knife-crime offences (6.7% of all knife crime). 156 offences were experienced by 72 repeat victim-offenders. Of the 610 unique victim-offenders, they committed 734 knife-crime offences (7.2% of all knife crime). 410 offences were committed by 145 repeat victim-offenders. Although this represents 4% of all knife crime, this equates to 55.8% of all knife-crime being committed by 23.7% of all victim-offenders.

### 5.2 The network structure of Thames Valley knife crimes

#### 5.2.1 Offender-victim networks

The offender-victim knife crime network is comprised of 9,261 unique actors spread across 7,044 knife crime incidents, with 5,020 and 4,550 unique offenders and victims, respectively. Moreover, only 22% (1,107) of offenders and 10% (442) of victims were affiliated with an OCG. As such, the network is comprised primarily of non-OCG-affiliated actors. As expected, there are no isolates within the network, as an actor cannot be both an offender and victim within the same dyadic pairing.

As shown in Table 2, the average indegree and outdegree centrality are proxies for the average number of offenders who have assaulted a victim and the average number of victims harmed by an offender. As such, knife crime victims have been accosted by 1.52 offenders, on average, whereas knife crime offenders have harmed an average of 1.37 victims. Importantly, we observed small but statistically significant differences in the outdegree and indegree centrality among OCG-affiliated and non-OCG-affiliated offenders and victims.

**Table 2:** Network and descriptive statistics (offender-victim).

Network Characteristics	Total / Means (standard deviation)	Range
Unique Actors/Nodes	9,261	-
Unique Offenders	5,020	-
Unique Victims	4,550	-
Offenders (Not OCG-affiliated)	3,908	-
Victims (Not OCG-affiliated)	4,108	-
Offenders (OCG-affiliated)	1,107	-
Victims (OCG-affiliated)	442	-
Isolates	0	-
Total Edges	6,879	-
Network Density	0	-
Indegree	1.52 (1.42)	1–64
Outdegree	1.37 (0.93)	1–13
Indegree (Not OCG-affiliated)	1.5 (0.35)	1–64
Indegree (OCG-affiliated)	1.76 (1.24)	1–9
Outdegree (Not OCG-affiliated)	1.3 (0.79)	1–13
Outdegree (OCG-affiliated)	1.62 (0.71)	1–10
Reciprocity	0.009	-
Modularity	0.997	-
Communities	2923	-

Independent samples *t-tests* revealed that OCG-affiliated offenders and victims possessed a higher average number of victims (1.62) and offenders (1.76) relative to their non-OCG-affiliated counterparts [(*t*(1370) = 8.94, *p* = <0.0001), and (*t*(575) = 4.2, *p* = <0.0001) respectively]. This reveals a key distinction between the frequency and intensity of knife crime offences: while non-OCG-affiliated offenders and victims may account for a larger proportion of knife crimes, OCG-affiliated offenders and victims are involved in a higher average number of knife crimes.

The distribution of outdegree and indegree centrality reveals that 78.1% and 72.9% of all offenders and victims had only one victim and offender, respectively (see Table 3). Furthermore, 66.6% and 60% of OCG-affiliated offenders and victims had a single victim and offender while 81.3% and 74.3% of non-OCG-affiliated offenders and victims had a single victim and offender. This suggests that the overwhelming majority of knife crimes are one-off events between two individuals.

**Table 3:** Distribution of in/out degree centrality (offender-victim).

Degree Centrality	Outdegree Total	Outdegree Non-OCG	Outdegree OCG	Indegree Total	Indegree Non-OCG	Indegree OCG
1	3920 (78.1%)	3179 (81.3%)	741 (66.6%)	3318 (72.9%)	3053 (74.3%)	265 (60%)
2	699 (13.9%)	492 (12.6%)	207 (18.6%)	674 (14.8%)	577 (14%)	97 (21.9%)
3	224 (4.5%)	146 (3.7%)	78 (7%)	307 (6.7%)	265 (6.5%)	42 (9.5%)
4	75 (1.5%)	37 (0.9%)	38 (3.4%)	129 (2.8%)	113 (2.8%)	16 (3.6%)
5	59 (1.2%)	36 (0.9%)	23 (2.1%)	55 (1.2%)	43 (1%)	12 (2.7%)
6	18 (0.4%)	6 (0.2%)	12 (1.1%)	37 (0.8%)	32 (0.8%)	5 (1.1%)
7	10 (0.2%)	6 (0.2%)	4 (0.4%)	14 (0.3%)	10 (0.2%)	4 (0.9%)
8	8 (0.2%)	4 (0.1%)	4 (0.4%)	7 (0.2%)	7 (0.2%)	0 (0%)
9+	7 (0.1%)	2 (0.1%)	5 (0.4%)	9 (0.2%)	8 (0.2%)	1 (0.2%)
**Total**	**5020 (100%)**	**3908 (100%)**	**1112 (100%)**	**4550 (100%)**	**4108 (100%)**	**442 (100%)**

**Fig 2:**
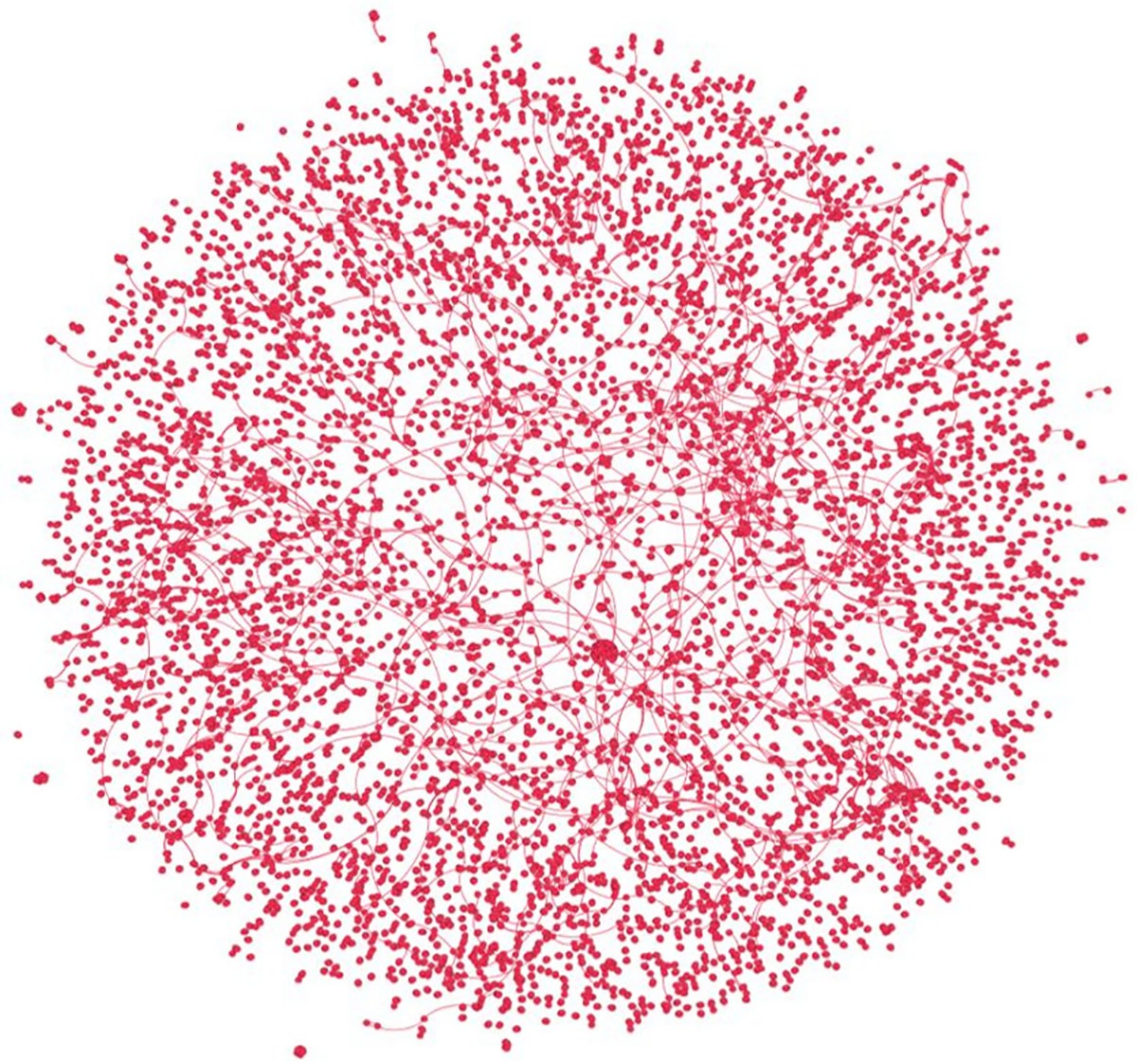
Offender-victim knife crime network.

Like many other criminal networks, the offender-victim knife crime network is markedly diffused, with a network density of 0 (note: the network density was rounded to 0). For the sake of comparison, the density of a dark web opioid distribution market studied by Duxbury and Haynie [66] was 0.002, while Jihadi networks examined by Krebs [67] and Morselli, Giguere, and Petit [68] ranged from 0.2 to 0.4. Furthermore, this network possessed a modularity of 0.997, with 2,923 identifiable communities. This suggests a high level of network fragmentation as nodes within the network exhibited clustering with respect to their given node grouping (see Fig 2).

In general, the structure of the offender-victim knife crime network is dyadic, as components with two nodes accounted for a majority (65.3%) of all components and a plurality (41.2%) of all nodes within the network. Furthermore, triads accounted for the second largest component group, representing 17.5% and 16.6% of all components and nodes, respectively. Indeed, knife crimes in Thames Valley are dyadic: one-off crime events between two individuals. This, moreover, explains the marked fragmentation as this network is comprised of many two-node subgroups while lacking many large multi-node components (Fig 2).

However, the fragmentation and dyadic composition of the offender-victim knife crime network may be a by-product of the relationship between the individuals involved. Our data show that 34.7% (2,444) and 33.1% (2,330) of knife crime incidents in this network involved strangers and acquaintances, respectively. While many law enforcement databases struggle from missing data, this particularly database was mainly complete with 97% of knife crime incidents possessing the relationship between the victim and offender. Nevertheless, it is important to acknowledge that as stranger violence is more likely to be reported, this finding may nevertheless be subject to a recording bias. Equally important are the criminal and victimisation histories of these offenders and victims (see Table 4). While a plurality (25.8%) of offenders had no prior criminal offending, a majority (52.9%) of these offenders had no prior victimisations. Similarly, 60.2% and 51.1% of victims had no prior offending or victimisations. However, knife crime offenders are more likely to have a criminal record, with 74.2% having committed at least one prior offence.

**Table 4:** Distribution of previous offences and victimisations (offender-victim).

No. Previous Offs/Vics	Frequency of Offenders (Offences)	Frequency of Offenders (Victimisations)	Frequency of Victims (Offences)	Frequency of Victims (Victimisations)
0	1296 (25.8%)	2654 (52.9%)	2739 (60.2%)	2325 (51.1%)
1	600 (12%)	881 (17.5%)	497 (10.9%)	590 (13%)
2	458 (9.1%)	579 (11.5%)	260 (5.7%)	544 (12%)
3	301 (6%)	320 (6.4%)	184 (4%)	253 (5.6%)
4	262 (5.2%)	185 (3.7%)	130 (2.9%)	222 (4.9%)
5	195 (3.9%)	120 (2.4%)	112 (2.5%)	154 (3.4%)
6	177 (3.5%)	79 (1.6%)	69 (1.5%)	114 (2.5%)
7	156 (3.1%)	45 (0.9%)	62 (1.4%)	70 (1.5%)
8	154 (3.1%)	44 (0.9%)	47 (1%)	53 (1.2%)
9	102 (2%)	26 (0.5%)	40 (0.9%)	36 (0.8%)
10–15	511 (10.2%)	56 (1.1%)	199 (4.4%)	126 (2.8%)
16–20	243 (4.8%)	19 (0.4%)	66 (1.5%)	34 (0.7%)
21–29	274 (5.5%)	8 (0.2%)	65 (1.4%)	19 (0.4%)
30–39	131 (2.6%)	4 (0.1%)	42 (0.9%)	3 (0.1%)
40+	160 (3.2%)	-	38 (0.8%)	7 (0.2%)
**Total**	**5020 (100%)**	**5020 (100%)**	**4550 (100%)**	**4550 (100%)**

#### 5.2.2 OCG-to-OCG network

**Fig 3:**
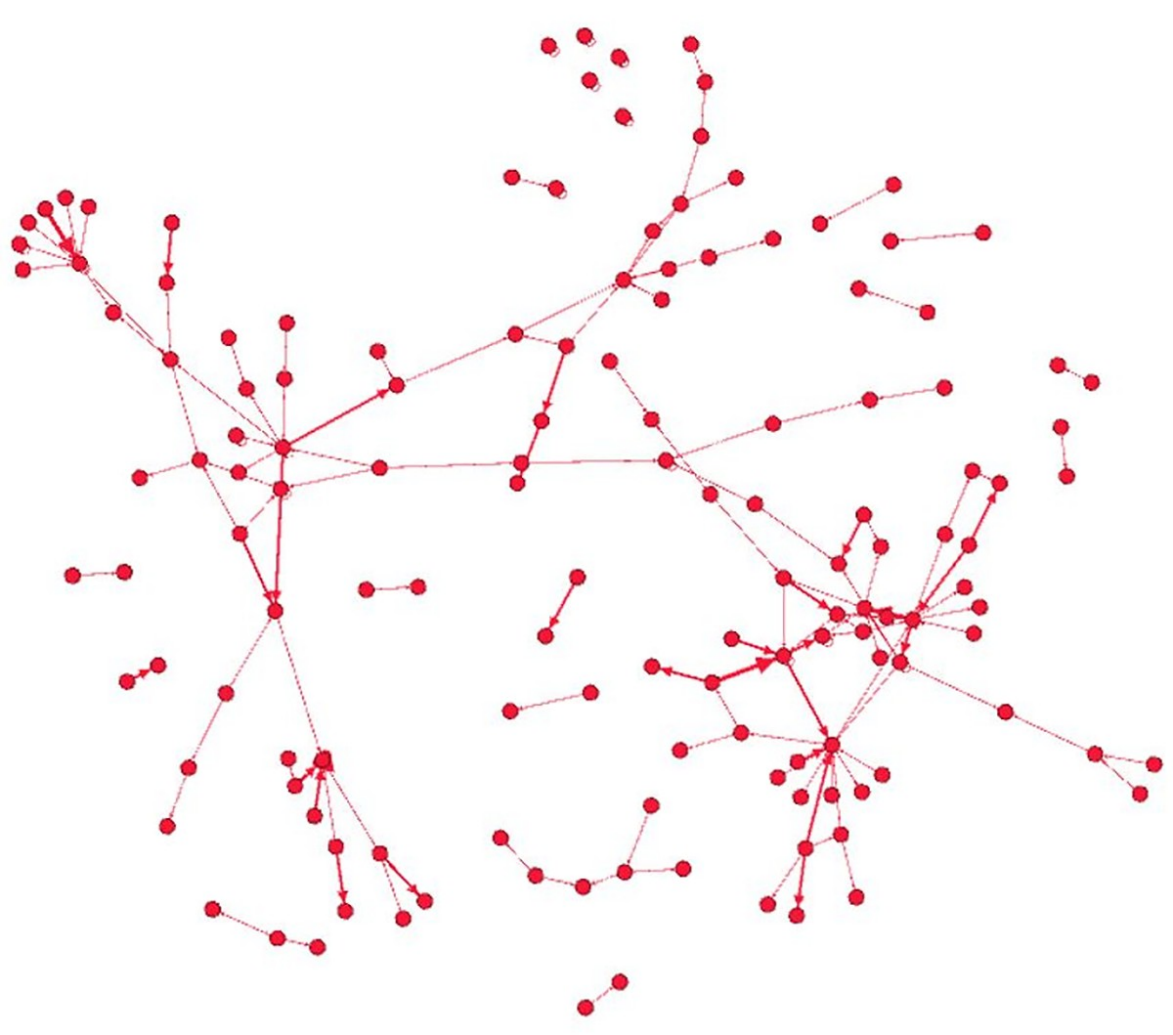
OCG-to-OCG knife crime network.

The OCG-to-OCG knife crime network is comprised of 141 unique OCGs, including 97 unique offending OCGs and 86 unique victimised OCGs (see Table 5 and Fig 3 for more details). These are spread across 209 knife crime incidents. 17.8% of all incidents involved an offender and victim from the same OCG, so we see that that in the majority of cases of when OCG members use knives as an assault weapon, they are directed at rival OCGs.

**Table 5:** Network and descriptive statistics (OCG-OCG).

Network Characteristics	Mean or Total	Range
Unique OCGs/Nodes	141	-
Unique Offending OCGs	97	-
Unique Victimised OCGs	86	-
Isolates	5	-
Total Edges	159	-
Network Density	0.008	-
Indegree	1.85	1–9
Outdegree	1.64	1–6
Reciprocity	0.02	-
Modularity	0.796	-
Communities	27	-

As per the average indegree centrality (Table 5), victimised OCGs are harmed by 1.85 OCGs, on average, whereas offending OCGs commit harm against 1.64 OCGs, on average. In short, it does not appear that inter-OCG knife crimes occur at a high frequency among a bevy of OCGs. This is corroborated by the distribution of outdegree and indegree centrality measures, where 62.9% of offending OCGs harmed only one OCG, while 60.5% of victimised OCGs were harmed by a single OCG. Only 22.7% and 22.1% of offending and victimised OCGs harmed or were harmed by two OCGs, respectively. In addition to a low reciprocity of 0.02, it appears that OCG-related knife crimes, like those committed by persons, are one-off events that rarely beget retaliation. To this point, only 2.9% (6) of all cases were tied to retaliation, and this network is not dyadic, as 73% (103) of all nodes are housed within a single component.

#### 5.2.3 Retaliations within the offender-victim network

Of the 7,044-recorded knife crime incidences in this network, 0.94% (66) were retaliations where actor *j* harmed actor *i* following actor *i*’s initial attack on actor *j*. As such, we found 33 retaliatory incidents, representing 33 mutual dyads in the network. This small number of retaliations is ultimately reflected in the low reciprocity of 0.009. This finding supports the established trend regarding the one-off nature of Thames Valley knife crimes.

Based on this small sample, we were able to determine that retaliations occurred within 16.5 days (or 395.15 hours) after the initial knife crime. However, this number should be cautiously considered as 12 of the 33 retaliatory attacks were instantaneous, occurring immediately (within a minute of the initial assault) following the initial attack. Importantly, only 12% of offenders and victims were affiliated with an OCG. As such, knife crime retaliations are not primarily a gang-related phenomenon. Of the 33 recorded retaliations, only three involved both an OCG-affiliated offender and victim. However, offenders who engaged in the initial and retaliatory knife crime were more likely to have an extensive criminal history relative to the rest of the offenders within the network. That is, 38% and 29% of the initial and retaliatory offenders had committed nine or more prior offences, respectively.

## 6. Discussion

### 6.1 Knife crime is male-dominated, with young adults between 16–34 and a criminal background—but not gang-related. The same can be said about victims of knife crime

Our data provide information on the nature and structural composition of knife crimes in the TVP Force Area. If our results are generalizable, then UK knife crime networks typically involve strangers, both of whom have a criminal history or prior victimisation, but are not gang affiliated, as over 81% of offenders and 92.2% of victims were found to be unaffiliated with any organised crime groups. We contextualise these findings more broadly within two distinct layers: individuals and networks.

The characteristics of victims and offenders bear marked similarities. That is, the age range, gender and ethnicities of these two groups are more similar than they are different, with males between the ages of 16 and 34 constituting the majority of victims and offenders. In terms of ethnicity, we found little difference between victims and offenders, with White (Northern Europeans) representing 72% and 66% respectively. Asian and Black ethnicities, however, are over-represented in the data, compared to their overall population trends in the TVP area. In general, the demographic similarities between victims and offenders provide some support for the existence of a victim-offender overlap in knife crimes [9], although with some variation in frequency and scope.

While a plurality (25.8%) of offenders had no prior criminal offending, a majority (52.9%) of these offenders have no prior victimisations. Similarly, 60.2% and 51.1% of victims had no prior offending or victimisations. The preponderance of first-time victims points to the peculiar nature of knife crimes in Thames Valley as most victims are generally not involved in crime of any sort. However, the offender-victim network indicated that the majority of offenders were known to the police from prior offences (74.2%). Nearly half of those victimised were repeat victims (47.1%). If these police records represent true population means of knife crime offenders and victims, then knife crime is correlated with criminal behaviour more broadly. This deviates from common assaults and less severe forms of violence, which is more likely to be a result of contextual factors (e.g. alcohol) rather than a crime committed by a seasoned offender. Knife crime is different: those who carry knives, and those who use them to commit crimes (and get caught), are known offenders. Even the victims are usually known to the police, with 39.8% having a criminal record, and 38.9% having been injured in the past. These trends are not very different from those reported in earlier studies [5, 31, 33].

Finally, the literature points to gang involvement as one of the main correlates of violent crime. However, the TVP data suggest otherwise, with under 19% of offenders and 7.8% of victims marked by an association with a recognized gang or organized crime. These figures support Massey et al. [69] who found that only 21% of knife homicides in London were gang related. More broadly, these findings debunk much of what is portrayed in the media: drug gangs escalating the risk of knife crime by using violence to exploit and obtain territory [70]. Indeed, it appears that TVP knife crimes are not usually gang related, and in fact experienced mostly by young and non-criminals.

### 6.2 Social networks of knife crime are usually dyadic and non-reiterative

The construction of both an offender-victim and OCG-to-OCG network offers unique insight into the structural composition of Thames Valley knife crimes on both an individual and organizational level. The offender-victim network was constructed from 7,044 of the 10,099 knife crimes, representing 69.7% of all incidents, including 5,020 (69.4%) unique offenders and 4,550 (65.9%) unique victims. Despite their involvement in the majority of knife incidents, non-OCG affiliated offenders and victims have fewer unique victims and offenders than their OCG-affiliated counterparts. This small but statistically significant difference reveals a key distinction in terms of the frequency and intensity of knife crimes: while non-OCG-affiliated offenders and victims account for a greater proportion of police-recorded knife criminality, OCG-affiliated victims and offenders are more frequently involved in these offences.

Moreover, 78% of all offenders and 73% of all victims had only one victim and offender, but when broken down into OCG and non-OCG-affiliated offenders and victims, 67% and 60% of OCG-affiliated offenders and victims had a single victim and offender, while 81.3% and 74.3% of non-OCG-affiliated offenders and victims had a single victim and offender. This indicates that an overwhelming majority of knife crimes are one-off events between two individuals. This is also reflected in the distribution of component sizes. Indeed, the victim-offender network is predominantly dyadic, where 65.3% of all components, comprising 41.2% of all nodes, are dyads. There is a distinct absence of many large multi-node components.

These findings seemingly run contrary to much of the co-offending network literature in the United States, which often reports that violent crime across high-crime communities occurs within small, identifiable networks of individuals actively engaged in criminal and delinquent behaviour [10, 53, 71]. Papachristos et al. [72] found in a high-crime neighbourhood in Boston that 85% of all gunshot injuries occurred within a single network containing only 763 individuals, two-thirds of whom were either gang members or had been arrested in the months leading up to their victimisation. These differences may be explained by cultural distinctions between gangs across the Atlantic, but they can also be the product of structural differences in geographic locales [73]. Studies by Papachristos et al. [10, 74] presented results from densely populated U.S. cities with well-established gang and gun violence. The knife crime study presents data arising from three UK diverse counties, which provide heterogeneity in landscape (rural and urban), culture and crime trends. As such, findings from studies of criminal networks in the U.S. [75, 76] cannot be duplicated for criminal networks in the UK; additional research is required to expound these disparities.

However, an equally likely explanation for this discrepancy may lie in the nature of the co-offending network examined. In general, U.S.-based studies deal predominantly with broader co-offending networks that are larger and subject to greater network density. For example, studies by Papachristo et. al [10, 55] and Papachristos and Wildeman [71] which examined gun violence networks in the U.S. included all arrest and co-arrests. Being more broadly defined, these networks are much larger, with more connections between victims and offenders relative to the network examined in this study. Furthermore, as this network is defined only by victims and offenders involved in knife crimes, it is unlikely to have a network density like in the aforementioned studies.

It is, however, arguable that the idiosyncrasies of the Thames Valley knife crime network are a consequence of the attributes of the individuals involved. To this extent, 34.7% and 33.1% of all knife crime incidents involved strangers and acquaintances, respectively. As the offenders and victims are strangers and acquaintances who have rarely or never associated with one another, it is unlikely that they will meet again following their altercation.

### 6.3 Tit-for-tat is uncommon

Finally, we find little evidence for retaliatory knife crime. We assumed that retaliatory assaults are common. Yet the SNA indicated otherwise: given the dyadic relationships in terms of knife crime that takes place primarily between unfamiliar strangers, the likelihood of a repeated transgression between the same parties is low: only 33 retaliatory incidents were identified. The overwhelming majority of knife crimes (99.61%) begin and end with the initial knife crime event. This finding can be explained by the criminal involvement of the offenders and the victims: the majority of them are not gang-affiliates, who are more likely to retaliate.

The literature is mixed on this issue of retaliation. Some find support for tit-for-tat [77–79], while others do not, finding no evidence of retaliation when the incident was perpetrated by a stranger or someone the victim barely knew [10]. Our findings support the latter perspective, given the rarity of this recording. The number of incidents is too low for valid forecasts, at least in the context of the Thames Valley. Nevertheless, offenders who participated in retaliatory knife incidents were more likely to have an extensive criminal history relative to the rest of the offenders within the network. That is, 38% and 29% of the initial and retaliatory offenders had committed nine or more prior offences, respectively. This, in part, suggests that retaliatory knife crimes are between actors with extensive criminal histories. This finding provides an important opportunity for proactive policing, whereby preventative tactics can be employed to deter high-risk retaliators from committing an offence following knife crime victimisation [80].

### 6.4 OCGs and gangs play a limited role in knife crime

The OCG-to-OCG network is comprised of 208 knife crime incidents, 82.2% of which involved different groups. Moreover, victimised OCGs were harmed by 1.85 OCGs while offending OCGs harmed 1.64 rivals. The majority of OCGS—62.9% and 60.5% of offending and victimised OCGS—harmed and were harmed by only one OCG, respectively. In addition to a low reciprocity of 0.02, it appears that OCG-related knife crimes, like those in the offender-victim network, remain one-off events that rarely beget retaliation. These findings contradict popular claims of OCGs driving UK knife crimes.

However, unlike the offender-victim network, the OCG-to-OCG network is not dyadic, with 73% of all nodes being housed within a single component. This is particularly telling as it indicates that OCG-related knife crimes are not densely reciprocated incidents between a small contingent of rival OCGs, but crime events that sparsely tie together an assortment of OCGs. Taken together, these results indicate that knife crimes perpetrated by TVP OCGs are not only rare, but also generally unreflective of the violent turf wars that characterise North American OCG and gang activities [77, 81].

### 6.5 Policy implications: Pre-emptive prevention measures with known offenders

From a practical perspective, dramatically limiting the number of potential offenders and victims—or both—from the overall population of people who live or visit a jurisdiction creates a finite pool of individuals for law enforcement agencies to control. Indeed, the relative contribution of each offender to overall knife crime is not shaped in a ‘power few’ formation [82], with a relatively small number of individuals responsible for a disproportionate number of incidents [83]. Still, some clustering does take place. Our analyses suggest that about one-fifth of repeat offenders committed about 42% of all the knife crime [84]. Nevertheless, they are known to the police (by virtue of being repeatedly arrested), and there are still some violent offenders who engage more regularly in crime than others. These data distributions present targeting opportunities—for example through focused deterrence approaches [85, 86].

The most immediate policy implication, however, concerns data and recording practices. As discussed by Massey et al. [69], we have also experienced data issues that hinder both forecasting as well as prevention of knife crime: the forced localisation of the data to the spatial boundaries of individual police forces; the ‘free text’ nature of many data points (for instance, the very recording of a knife); or the accuracy of data—all equally remain a source of concern in this line of research. Crime recording systems that do not allow for reasonable retrieval of historic data pose challenges. Equally, the feedback of data on the whereabouts of suspects, persons of interest and potential victims of knife crime (or violence more broadly) to field officers is sporadic and limited. Thus, more valid, reliable and available data can assist in understanding the phenomenon, as well as guide us toward effective preventative or suppressive initiatives.

As it relates to policy and law enforcement intervention, there is a need to separate gangs from knife crimes in order to tackle both problems more effectively. Whilst most policing approaches deal with the problem of gangs and knives as one-in-the-same, these findings suggest their independence from one another. Moreover, given the low density of the knife crime network, it is evident that knife crimes are one-on-one events. As the network is fragmented, it may be difficult for law enforcement to craft a targeting strategy which focuses on key actors within the network. However, this insight may prove useful as law enforcement should pivot away from interventions which target gangs to a generalized approach which discourages carrying knives in public. With that said, in order to identify those at greatest risk of high-harm knife crimes, police data recording systems must reduce duplicated entries and include options to record how a knife was used. With these improvements, a more valid assessment of knife crime harm could be conducted.

### 6.6 Future research implications

Overall, the characteristics of habitual knife crime offenders or repeat knife crime victims are not different from ‘one-off’ knife offenders and victims. We sense that our data are limited and therefore we are unable to trace discrete features of these subgroups. With additional information, classification of violent offenders is more likely. For example, we know that differences between offenders are often explained by situational, psychosocial or ecological factors—however these data points were beyond our reach. Future research should consider richer data and more fit actuarial models to forecast involvement of repeated knife crime. Such analysis may debunk one possible explanation for the habitualness we find in our data: a disproportionate attention on ‘likely suspects’ on the part of law enforcement [51]. While it may not be possible to use the current data to accurately forecast likely offenders, future research should consider how network-level data can be applied for the purposes of identifying knife crime risk. Such risk factors can be further developed to form the foundation of an actuarial forecasting tool for knife crimes. To this extent, this study has demonstrated the utility of social network analysis in determining the underlying structure of a knife crime network. Future research should consider utilizing social network analysis in order to replicate the findings of this study (e.g. network density, modularity, etc.).
